# Multiplex Hybrid Antigen-Capture LC-MRM Quantification in Sera and Nasal Lining Fluid of AZD7442, a SARS-CoV-2-Targeting Antibody Combination

**DOI:** 10.1021/acs.analchem.2c01320

**Published:** 2022-10-21

**Authors:** Ruipeng Mu, Yue Huang, Jerome Bouquet, Jiaqi Yuan, Robert J. Kubiak, Eric Ma, Sami Naser, William R. Mylott, Omnia A. Ismaiel, Aaron M. Wheeler, Rebecca Burkart, Diego F. Cortes, James Bruton, Rosalinda H. Arends, Meina Liang, Anton I. Rosenbaum

**Affiliations:** † Integrated Bioanalysis, Clinical Pharmacology and Safety Sciences, R&D, AstraZeneca, South San Francisco, California 94080, United States; ‡ Clinical Pharmacology and Quantitative Pharmacology, Clinical Pharmacology and Safety Sciences, R&D, AstraZeneca, Gaithersburg, Maryland 20878, United States; § Research and Development Biologics by LC-MS/MS, PPD Laboratories, Richmond, Virginia 23230, United States; ∥ Faculty of Pharmacy, Zagazig University, Zagazig 2, Zagazig, Egypt

## Abstract

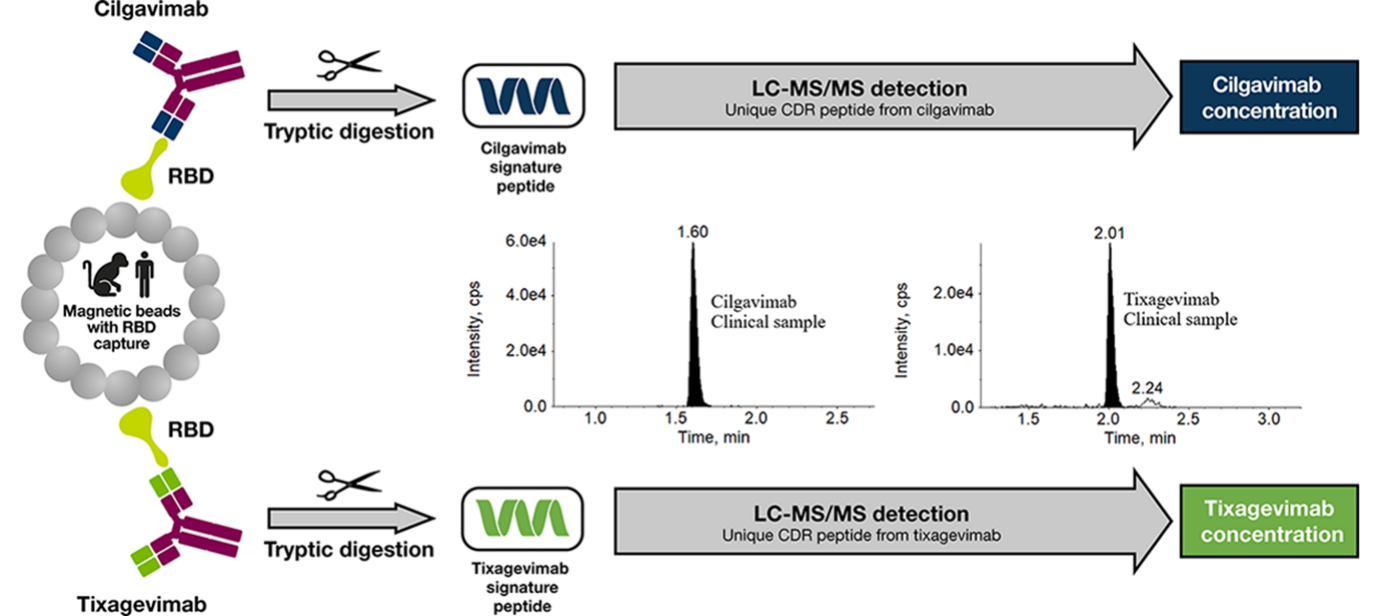

AZD7442 (tixagevimab [AZD8895]/cilgavimab [AZD1061]) is a monoclonal antibody (mAb) combination in development for the prevention and treatment of coronavirus disease 2019. Traditionally, bioanalysis of mAbs is performed using ligand binding assays (LBAs), which offer sensitivity, robustness, and ease of implementation. However, LBAs frequently require generation of critical reagents that typically take several months. Instead, we developed a highly sensitive (5 ng/mL limit of quantification) method using a hybrid LBA-liquid chromatography coupled with tandem mass spectrometry (LC-MS/MS) approach for quantification of the two codosed antibodies in serum and nasal lining fluid (NLF), a rare matrix. The method was optimized by careful selection of multiple reaction monitoring, capture reagents, magnetic beads, chromatographic conditions, evaluations of selectivity, and matrix effect. The final assay used viral spike protein receptor-binding domain as capture reagent and signature proteotypic peptides from the complementarity-determining region of each mAb for detection. In contrast to other methods of similar/superior sensitivity, our approach did not require multidimensional separations and can be operated in an analytical flow regime, ensuring high throughput and robustness required for clinical analysis at scale. The sensitivity of this method significantly exceeds typical sensitivity of ∼100 ng/mL for analytical flow 1D LBA-LC-MS/MS methods for large macromolecules, such as antibodies. Furthermore, infection and vaccination status did not impact method performance, ensuring method robustness and applicability to a broad patient population. This report demonstrated the general applicability of the hybrid LBA-LC-MS/MS approach to platform quantification of antibodies with high sensitivity and reproducibility, with specialized extension to matrices of increasing interest, such as NLF.

## Introduction

AZD7442 (tixagevimab [AZD8895]/cilgavimab [AZD1061]) is a combination of two long-acting immunoglobulin G (IgG) kappa monoclonal antibodies (mAbs) that neutralize SARS-CoV-2 by simultaneously binding to distinct epitopes on the viral spike protein receptor-binding domain (RBD), blocking interaction with human angiotensin-converting enzyme 2, and preventing viral entry into host cells. The PROVENT study reported significant efficacy and safety in the prevention of symptomatic COVID-19.^1^ Compared with the progenitor antibody combination (COV2-2196 and COV2-2130) isolated from the B cells of individuals with prior SARS-CoV-2 infection, tixagevimab and cilgavimab each contain amino acid modifications (YTE and TM) in the fragment crystallizable (Fc) regions to prolong their serum half-lives and to abrogate Fc-mediated effector functions, respectively.^2^

To support accelerated preclinical and clinical development of AZD7442, to address the urgent needs of the pandemic, robust and highly sensitive bioanalytical methods for the quantification of tixagevimab and cilgavimab in sera and nasal lining fluid (NLF) from humans and cynomolgus macaques were developed simultaneously and validated in accordance with relevant regulatory guidelines.^3,4^ Traditionally, mAb bioanalysis has been performed by ligand binding assays (LBAs),^4,5^ which offer great robustness, sensitivity, and ease of implementation. However, key reagents, such as anti-idiotype (anti-ID), are critical for quantification of human (or humanized) antibodies from human matrix. The generation of anti-ID antibodies typically takes several months. Therefore, we selected liquid chromatography coupled with tandem mass spectrometry (LC-MS/MS) for the development of bioanalytical methods that could differentially quantify tixagevimab and cilgavimab without the need to generate selective critical reagents, accommodating required ultra-accelerated study timelines and enabling high sensitivity quantification in NLF, a rare matrix.

In particular, we significantly optimized the method for the quantification of tixagevimab and cilgavimab in human NLF samples to help determine pharmacologically active concentrations at the expected initial site of SARS-CoV-2 infection.^6^ Analyses of drug concentrations in NLF had been hindered by the lack of convenient and reliable sampling methods, and the extremely low analyte concentrations associated with traditional sample collection methods. This resulted in broad ranges reported for the partition ratio of biopharmaceuticals into the nasopharynx.^7−9^ For AZD7442, by using the novel synthetic absorptive matrix (SAM) technologies^10^ in combination with a highly sensitive and selective bioanalysis method, sufficient collection volumes were obtained to allow reliable measurement of low target analyte concentrations.

In this report, we describe the strategy and approaches taken toward the development of a quantitative validated method for the assessment of AZD7442 pharmacokinetics (PKs) in the sera of humans and cynomolgus macaques, and a fit-for-purpose method for quantification of AZD7442 in NLF. The resulting method combines several breakthrough developments, eliminating the need for anti-ID capture antibodies and enabling robust, high sensitivity, multiplex quantification of any antibody combination, and offers an innovative bioanalytical strategy for antibody combination therapy with aggressive development timelines while withstanding potential matrix interferences.

## Experimental Section

**Figure 1:**
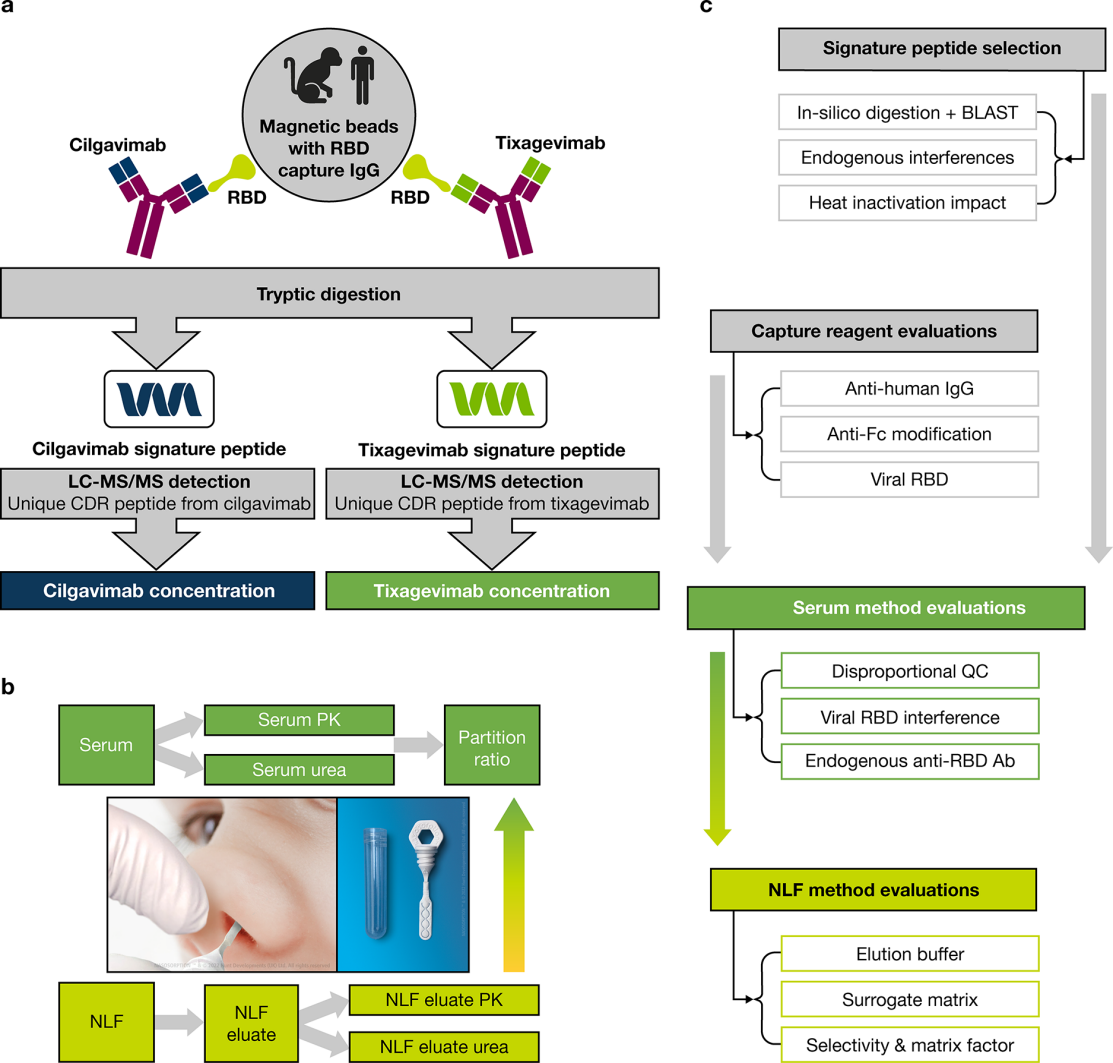
Schematic of the methodology employed and method optimization for the LBA-LC-MS/MS assays for the quantification of AZD7442. (a) The overall quantification approach from either serum or NLF. (b) The nasal pharmacokinetic assay for AZD7442 is set up to measure both the AZD7442 concentration and the urea concentration in NLF for the normalization of the data. (c) Streamlined strategic experimental design for rapid development of serum and NLF assay methodology. Images in Figure 1b used with permission from Mucosal Diagnostics, including copyright statement; NASOSORPTION & © 2022 Hunt Developments (UK) Ltd. All rights reserved.

The methodology surrounding the identification and quantification of tixagevimab and cilgavimab in serum and NLF is illustrated in Figure 1a and b.

### Materials and Methods

To cover the complex bioanalytical support for the serum and nasal concentration of AZD7442 in a broad spectrum of human population, a range of assays were developed, evaluated, and deployed. This includes the core methods with RBD immunocapture coupled with LC-MS/MS for the measurement of AZD7442 concentration, as well as the urea concentration method for data normalization and serological assays for population endogenous interference characterization. Relevant methods are summarized in Table 1 along with critical methodological information. More detailed information is provided in the Supporting Information.

**Table 1:** Summary of Methods Employed in this Study

Assay name	Assay type	Matrix	Dynamic range
Serum PK, Cynomolgus	LBA-LC-MS/MS, RBD capture	Cynomolgus serum	9–1,000 μg/mL
Serum PK, Human	LBA-LC-MS/MS, RBD capture	Human serum	0.3–30 μg/mL
Serum PK, Human	LBA-LC-MS/MS, anti-TM capture	Human serum	0.3–30 μg/mL
NLF PK	LBA-LC-MS/MS, RBD capture	Human NLF eluate	0.005–1.5 μg/mL
NLF urea	Enzymatic Colorimetric	Human NLF eluate	0–6.0 μg/mL
Serology assay (IgG)	Electro-chemiluminescence	Human serum	156–20,000 ng/mL
Serology assay (IgA)	Electro-chemiluminescence	Human serum	50.0–2,500 ng/mL
Serology assay (IgM)	Electro-chemiluminescence	Human serum	50.0–3,200 ng/mL

### Materials and Reagents

Tixagevimab and cilgavimab reference standards, RBD of SARS-CoV-2, and anti-TM antibody were provided by AstraZeneca (Gaithersburg, MD, USA). Stable isotope labeled internal standard peptides, ASGF-IS (ASGFTFMSSAVQWVR*, R* = ^13^C_6_, ^15^N_4_), and DVWM-IS (DVWMSWVR*) were supplied by Elim Biopharmaceuticals (Hayward, CA, USA).

Cynomolgus monkey and human sera were from BioIVT (Hicksville, NY, USA). High-capacity Magne streptavidin beads were purchased from Promega Corporation (Madison, WI, USA). Biotinylation kit EZ-LinkTM Sulfo-NHS-LC-Biotin, PierceTM Trypsin Protease (MS grade), NP-40 Surfact-Amps, and SMART IA digestion beads were from Thermo Fisher Scientific (Waltham, MA, USA). The noninvasive Nasosorption FXi nasal sampling device used for NLF collection was from Mucosal Diagnostics (Midhurst, UK). The urea assay kit was from Abcam (Cambridge, UK).

RapiGestTM SF surfactant, ACQUITY UPLC HSS, and BEH columns were from Waters (Milford, MA, USA). Tris(hydroxymethyl)-aminomethane-buffered saline (TBS) and TBS-Tween-20 (TBST) were from Boston Bioproducts (Ashland, MA, USA). Dulbecco’s phosphate-buffered saline (PBS) was from Mediatech (Manassas, VA, USA). All other general reagents, such as antihuman Fc antibodies, bovine serum albumin (BSA), DTT, and LCMS-grade solvents, were from Sigma-Aldrich (St. Louis, MO, USA) or VWR Scientific (Allison Park, PA, USA). High-grade water was obtained using a Milli-Q internal water purification system. Further details can be found in the Supporting Information.

### Instrumentation

For the LC-MS/MS method development, Nexera LC (Shimadzu, Kyoto, Japan) was used with a SCIEX Q-TOF 6600 or SCIEX triple quadrupole 6500+ mass spectrometer (Framingham, MA, USA). Data were acquired with SCIEX Analyst and analyzed with SCIEX PeakView and MultiQuant. For the assay validation and sample analysis, an LC PAL autosampler (CTC Analytics AG, Zwingen, Switzerland) with Agilent pumps and column heater (Agilent, Santa Clara, CA, USA) was used with either SCIEX triple quadrupole API-5000 or SCIEX 6500+. Data were acquired and analyzed with SCIEX Analyst software and PPD Assist LIMS (Richmond, VA).

For the colorimetric assay, analysis was performed on a SprectraMAX M3 microplate reader and optical density was measured at 570 nm (Molecular Devices, San Jose, CA). The serology assay was performed using a Meso Scale Discovery immunoassay (Newark, DE, USA).

### Signature Peptide Selection

Reference standards diluted with PBS or spiked in pooled human serum at various concentrations and blank human sera were used for signature peptide selection. For heat inactivation, spiked human serum samples were incubated in a 60 °C water bath for 1 h and compared against control samples. Briefly, samples (25 μL) were immunoprecipitated with the selected capture reagent immobilized on SMART IA beads (30 μL beads, 150 μL buffer, room temperature [RT], 2 h), washed multiple times with buffer to reduce serum background, mixed with internal standard (IS), and then digested (70 °C, 1.5 h). The digested samples were then separated on a Waters BEH C18 column on a Shimadzu LC coupled to a Sciex 6600 TripleTOF in full-scan and scheduled MS^2^ modes, or a Sciex 6500 triple quadrupole with a scheduled MRM list.

### Capture Reagent Evaluation

Different types of capture reagents were evaluated, including an anti-TM antibody, as well as the RBD of the SARS-CoV-2 spike protein (Figure 1c). Ultimately, RBD capture was selected to take advantage of high binding affinity, specificity, selectivity for the codosed mAbs, and availability of sufficient quantities of material. Moreover, the RBD capture approach ensured selectivity and sensitivity for the analysis of serum samples from cynomolgus monkeys and humans, and NLF samples from humans. This was followed by the development and validation of an anti-TM method, which was employed to evaluate the impact of vaccination and infection status on quantification.

Capture reagents evaluated included RBD, multiple clones of anti-TM antibodies, and commercially available antihuman Fc antibodies (clones HP-6001, 8c/6-39, GG-7, HP-6017), which were biotinylated and conjugated to SMART IA streptavidin beads. We also evaluated capture using SMART IA protein A/G beads. The conjugated beads were incubated with blank or reference standard-spiked human serum. The samples were then digested as previously described and separated on a Waters BEH C18 column on a Shimadzu LC coupled to a SCIEX 6600 TripleTOF in independent data acquisition with an inclusion list or scheduled MS^2^ mode.

### LBA-LC-MS/MS Methods

The serum LBA-LC-MS/MS method in human matrix with RBD capture was validated per relevant regulatory guidance^3,4^ with full characterizations of assay performance. Briefly, 20 μL of human serum sample were diluted with TBST/BSA buffer and mixed with RBD to coat high capacity Promega magnetic beads employed for the capture step (2 h, RT, shaking). After the capture step, the beads were washed thoroughly to reduce serum background. The beads were then transferred to the elution plate, denatured, reduced, and alkylated. Afterward, the mixture was digested with trypsin (37 °C, 2 h) and mixed with IS. The resulting solution was quenched with acid and passed through the multiscreen HTS filter plate before injection into the LCMS for detection.

The other LBA-LCMS methods employed for quantification from cynomolgus serum and NLF, as well as utilizing anti-TM capture, followed a similar approach as described above, with slight adjustments to accommodate differences in dynamic range and capture reagent. Method details are summarized in Table 1. All methods were validated or qualified for their purpose. Further method details can be found in the Supporting Information.

### NLF Elution Method

For the human NLF assay, patient samples were collected using a noninvasive Nasosorption FXi nasal sampling device, which uses a SAM swab to absorb NLF from the nasal mucosa (Figure 1b).^11^ The moist strip was removed from the device and added to 300 μL of elution buffer (PBS pH 7.4 with 1 mg/mL BSA and 1% NP-40 Surfact-Amps). Due to the limited collection volume and availability of blank NLF, a surrogate matrix consisting of 2.5% human serum in elution buffer was used for preparing calibration standards and quality control (QC) samples. A total of 19 individual human NLF samples were collected from healthy volunteers for the evaluation of method performance.

### NLF Urea Method

Due to expected variability from the collected NLF volumes from patients and from the extraction efficiency of the nasal swabs, urea concentrations were selected to correct/normalize the tixagevimab and cilgavimab concentrations in human NLF.^12,13^ Urea concentrations were determined with the urea assay kit, following manufacturer’s instructions over a concentration range of 0–6.0 μg/mL.

### Serology Methods for Vaccinated or Convalescent Sera

Anti-SARS-CoV-2 IgG, immunoglobulin M (IgM), and immunoglobulin A (IgA) antibody titers in human serum were measured with validated Meso Scale Discovery electrochemiluminescence methods.^14^ SARS-CoV-2 spike RBD was coated as a capture reagent for each method, and reactive IgG, IgM, and IgA were detected using detection antibodies specific to their respective Ig class.

## Results

**Figure 2:**
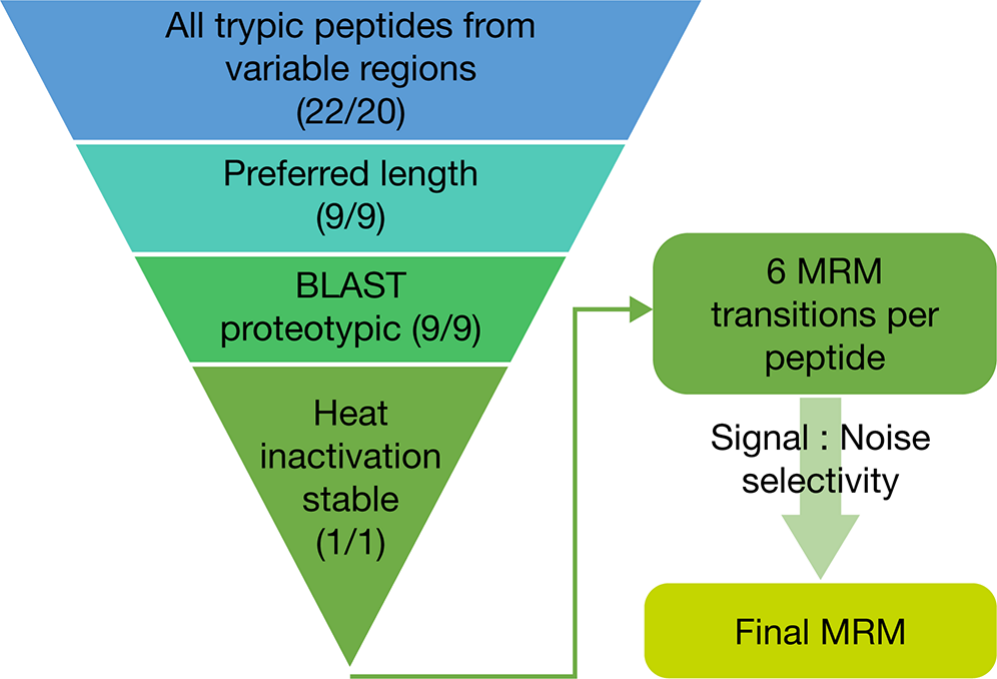
Peptide and MRM selection overview. Tryptic peptides from variable regions were selected based on peptide length (between 6 and 18 amino acids). Proteotypic peptides identified via BLAST search were then checked for stability under heat inactivation conditions. Subsequently MRM transitions were evaluated for signal-to-noise and selectivity enabling final selection.

In order to address the challenge of the lack of specific capture reagents, we focused on the optimization of several main parameters contributing to assay specificity, selectivity, sensitivity, and ultimately robustness. The overall process for method optimization is presented in Figure 1c. We focused our efforts on four key areas: signature peptide selection, capture reagent evaluation, and serum and NLF evaluations. Initially, careful selection of potential surrogate analyte peptides and MRMs was performed (Figure 1c, Figure 2).

In our method optimization, we focused on evaluation of tryptic peptides originating from complementary-determining regions (CDRs). A key characteristic for optimal peptide selection was a length of 6–18 amino acids. We also performed a BLAST^15^ search to ensure that the unique peptides selected were proteotypic, resulting in six detection peptide candidates and 43 MRM transitions for tixagevimab and cilgavimab. Neat tixagevimab and cilgavimab were digested with trypsin and analyzed using a high-resolution mass spectrometer (SCIEX 6600). Four unique peptides with the highest signal-to-noise ratio were further evaluated with a triple quadrupole mass spectrometer (SCIEX 6500+). Human serum samples spiked with tixagevimab and cilgavimab at various concentrations were used to optimize the chromatographic separation and MRM transitions (Figure 2); the selected peptides (MRM transitions) were ASGF (MRM 837.4/932.5) for tixagevimab and DVWM (MRM 539.7/864.5) for cilgavimab, as they provided optimal sensitivity and specificity/selectivity in both human and cynomolgus monkey sera. Moreover, we ensured that the methodology employed could withstand heat inactivation in the event that the samples originated from individuals infected with SARS-COV2 (Table S1). Furthermore, we evaluated six MRM transition peptides for signal-to-noise ratio and selectivity, leading to final MRM selection.

The criteria for selecting the capture reagent and detection peptides ensured that the assay met human serum assay requirements in terms of sensitivity, selectivity, and robustness. The method was later adapted to cynomolgus monkey serum, where the possibility of endogenous IgG interference was relatively low, and the assay quantification concentration was higher.

Two key aspects were evaluated during method development prior to validation: selecting the capture capacity, and evaluating the performance with disproportionate concentrations of tixagevimab and cilgavimab. To select the appropriate capture capacity, samples at the upper limit of quantification were prepared with different capture reagent: analyte ratios. Measured concentrations of tixagevimab and cilgavimab deviated by <20% from theoretical concentrations (Table S2a). In the clinical setting, there exists the potential for a theoretical interference between the RBD capture and AZD7442, including viral RBD and endogenous anti-RBD antibodies resulting from patient exposure to SARS-CoV-2. To mitigate the aforementioned possibilities, an excess amount of capture reagent (capture reagent to analyte ratio = nine) was utilized to ensure sufficient capture capacity in the clinical setting. To evaluate method performance if the codosed mAbs had a difference in clearance, resulting in different concentrations, especially at later time points, disproportionate QCs comprising of concentrations of tixagevimab and cilgavimab at ratios of 1:5 and 5:1 were evaluated. The results demonstrated that average percent differences from theoretical values were <10% for both analytes at each ratio, confirming no interference to quantification from codosed mAbs for up to a 5-fold differential in concentration (Table S2b).

The assays were successfully validated in human and cynomolgus monkey serum according to regulatory guidelines.^3,4^ Representative chromatograms for tixagevimab and cilgavimab quantification from human serum are shown in Figure S1a and S1b. Of note, the dynamic range of the serum methods employed (Table 1) was primarily based on the expected concentrations for the respective study samples,^1,2^ not limitations of assay sensitivity.

**Figure 3:**
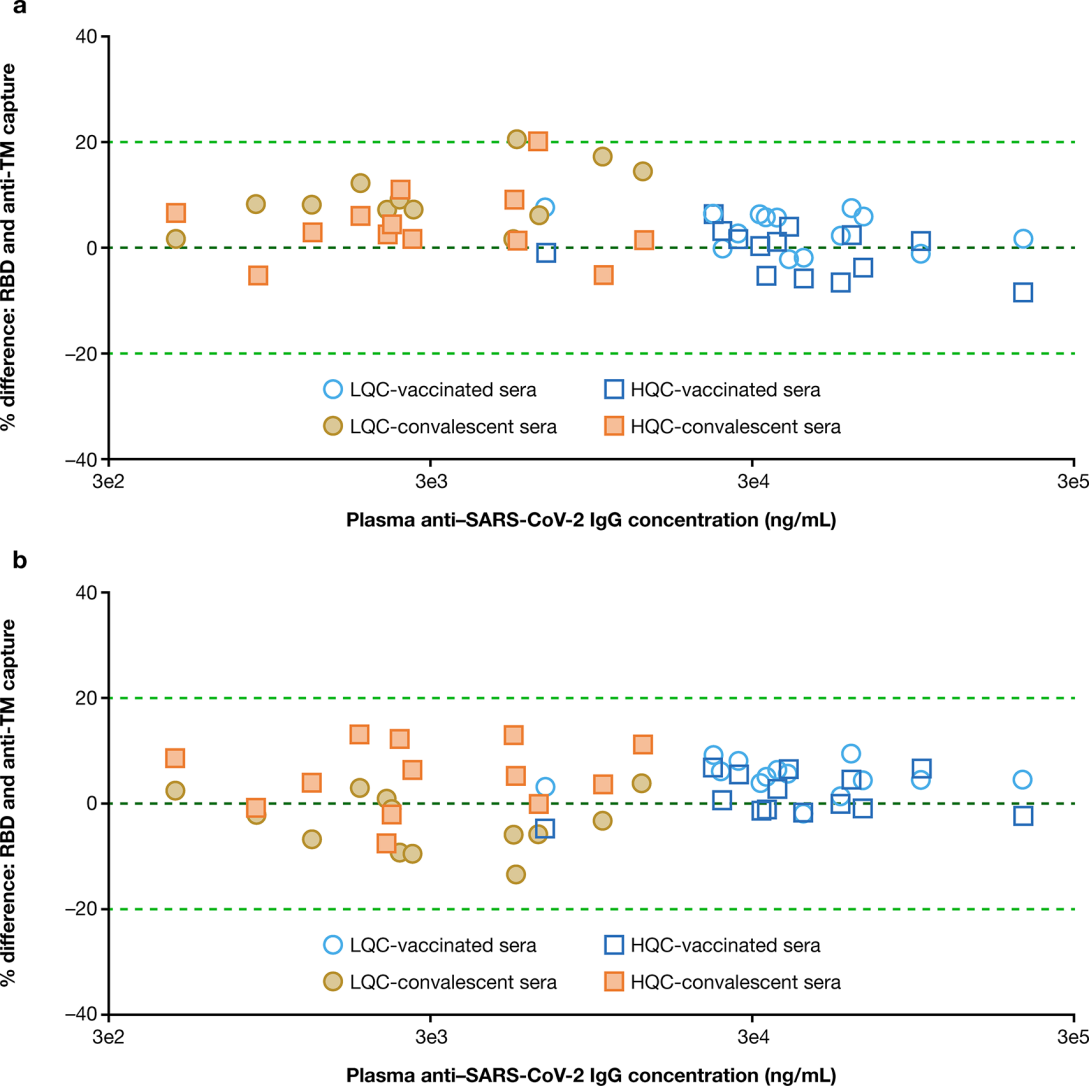
Percent difference in (a) tixagevimab and (b) cilgavimab quantification in convalescent sera and vaccinated sera between RBD and anti-TM antibody capture with different titer levels. For calculation of percent difference, measurements were taken in triplicate and mean concentrations calculated; percent difference was then obtained by dividing the difference of the two mean concentrations by the average value of the two. Accuracy and precision of both QC levels met assay acceptance criteria.

**Figure 4:**
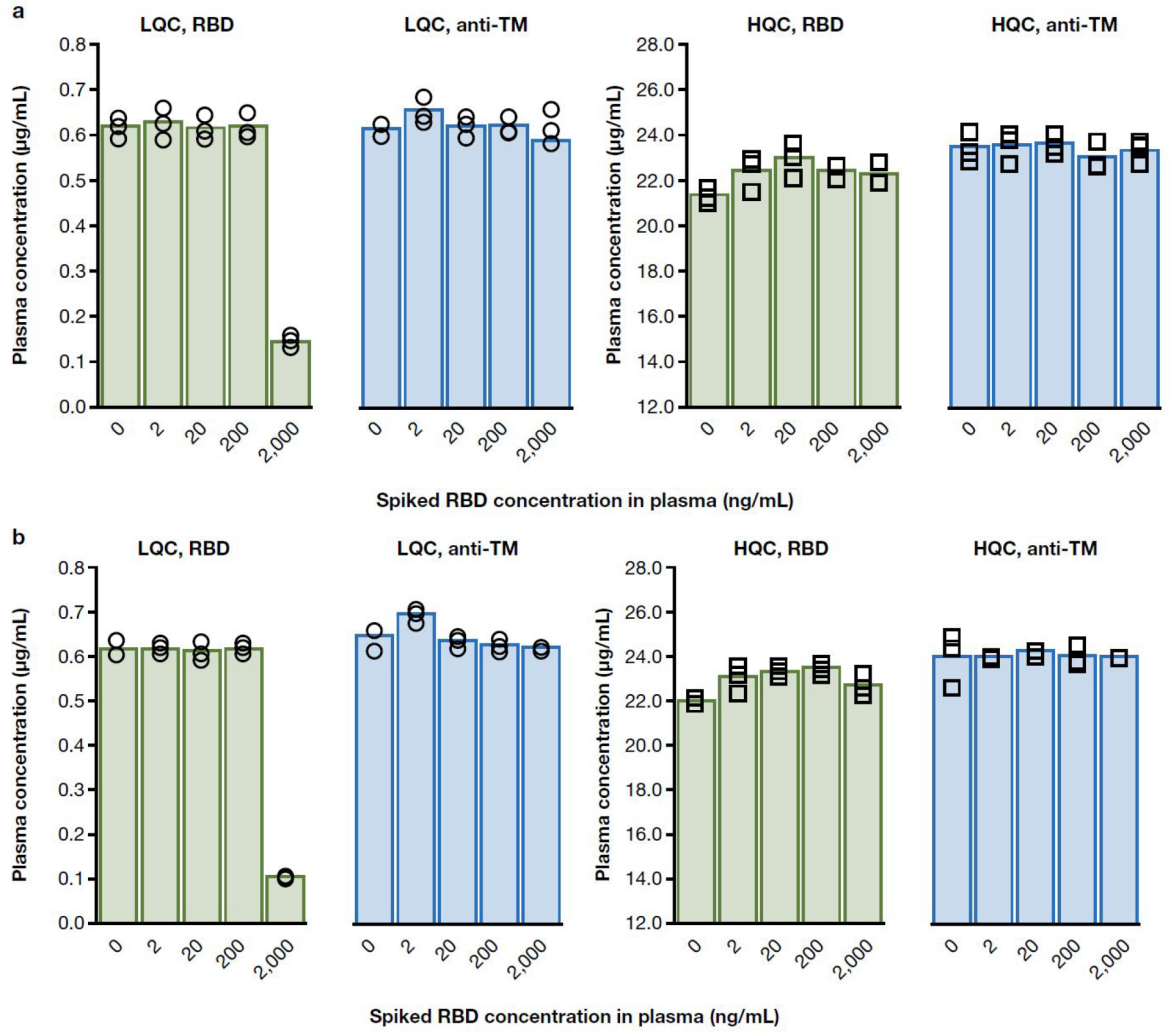
(a) Tixagevimab and (b) cilgavimab quantification using RBD and anti-TM antibody capture in human serum spiked with different levels of soluble RBD.

AZD7442 has been evaluated for both the prophylaxis and treatment of COVID-19. Therefore, it is important to assess whether the quantification of AZD7442 is impacted by the viral RBD or endogenous anti-RBD antibody from individuals upon infection or individuals that were vaccinated against SARS-CoV-2. As mentioned above, an assay with anti-TM antibody as the capture reagent was developed and validated in support of these evaluations. The two assays for quantification from human serum have an identical format except for the selection of capture reagent. To determine if endogenous anti-RBD antibody in COVID-19 convalescent or vaccinated patient sera could compete with tixagevimab and cilgavimab for binding to capture reagent in clinical sample analysis, two experiments were designed. In the first experiment, convalescent and vaccinated individual sera were used to prepare the low-level QC (LQC, 0.6 μg/mL) and high-level QC (HQC, 22.5 μg/mL). Quantification of tixagevimab and cilgavimab in convalescent and vaccinated patient sera using the RBD capture or anti-TM capture methods was comparable across a broad range of SARS-CoV-2 titers (Figure 3a and b), suggesting no interference from viral infection or vaccination-induced anti-RBD IgG. In the second evaluation, tixagevimab and cilgavimab were quantified at LQC and HQC levels with various concentrations of soluble RBD spiked. Quantification accuracy was impacted only when spiking 2,000 ng/mL soluble RBD to LQC samples and using RBD as the capture reagent (Figure 4a and b).

### Method Development for Human NLF

Several bioanalytical considerations were factored into the development of a PK assay for NLF. Collection of NLF has traditionally been performed using nasal washes or nasal swabs, which made it difficult to obtain suitable recovery of analytes, particularly where concentrations of target analytes are very low.^10^ Nasal swabs also require rotation within the nostril, which can be uncomfortable.^16^ Nasosorption is noninvasive and does not require rotation within the nostril, providing individuals with a more comfortable experience.^11^ Moreover, nasosorption using SAM strips enable the collection of more concentrated samples for analysis.^10^ The most critical step for NLF measurement is the elution of NLF from the SAM. Since the amount of NLF collected on each SAM nasosorption device can vary,^11^ three important bioanalytical considerations were evaluated: the performance of the assay with a wide range of matrix content in the elution buffer to account for potential differences in clinical sample collection; methods to maximize the elution efficiency of AZD7442 from the SAM; and appropriate normalization of concentrations obtained from clinical samples.

Since it is difficult to obtain appropriate amounts of blank NLF for use as a matrix blank, a surrogate matrix using serum with elution buffer was used. To evaluate the matrix effect with a wide range of matrix content in the elution buffer, two sets of QCs at different levels, prepared in 0% serum and 5% serum in elution buffer, mimicking NLF in buffer, were evaluated against the standard curve prepared in 2.5% serum in elution buffer. The recovery of tixagevimab and cilgavimab was evaluated at LQC (15.0 ng/mL) and HQC (1,150 ng/mL) levels. As shown in Table S3a, there was no apparent matrix effect for a serum concentration of 0–5%. The estimated amount of NLF in eluate is expected to be within this range, thus confirming the assay is fit for NLF AZD7442 quantification even if there were variance in the volume of NLF collected on the nasosorption device.

The initial elution buffer evaluated consisted of 50 mM **tris** (hydroxymethyl)aminomethane ([Tris] pH 7.5) with 1 mg/mL of BSA and 1% NP-40, which provided consistent and high absolute recovery (>80%).^17^ However, since the eluate was needed for the analysis of urea, amine containing buffers (Tris) had to be avoided. The elution buffer was therefore modified to 1X PBS (pH 7.4), with 1 mg/mL BSA and 1% NP-40. Elution recovery was evaluated for both analytes at the LQC and HQC concentration levels. The data in Table S3b demonstrate that the overall elution recovery across the concentration range for both tixagevimab and cilgavimab ranged from 92.2% to 97.2% and was highly consistent.

To normalize the AZD7442 concentration measured in the NLF eluted from the SAM, the urea concentration in NLF eluate was measured using a commercially available testing kit.^18^ While being in a generally predictable range, the urea concentration in NLF eluate may differ due to variability in the percent extraction of NLF from the strip by the eluate, and variability of the eluate volume used to extract the NLF. In the urea assay for NLF, a dilution of 10–20-fold was performed for all samples. The method was initially evaluated by analyzing NLF samples collected from 19 healthy donors, whose results were within the quantification range (data not shown), thus ensuring that the method fits the intended purpose.

The PK assay in human NLF was successfully qualified. Representative chromatograms for tixagevimab and cilgavimab quantification from human NLF are shown in Figure S1c and S1d. This method for analysis of tixagevimab and cilgavimab had a dynamic range of 5–1,500 ng/mL in eluate.

### Clinical Sample Analysis

Clinical sample data presented here are from a phase 1 clinical trial of the safety, tolerability, and PKs of AZD7442 in healthy adult participants with no prior history of COVID-19 and no prior receipt of a COVID-19 vaccine (clinicaltrials.gov identifier NCT04507256), as previously reported.^2^ This study is being conducted in compliance with the ethical principles originating in or derived from the Declaration of Helsinki, and in compliance with the International Confederation on Harmonization Good Clinical Practice Guidelines; all participants provided written informed consent before entering the study. Accordingly, the clinical data presented include only available preliminary data intended to support utility of the bioanalytical methods evaluated. Full results from the phase 1 trial will be published in due course. Overall, the assays presented were utilized in support of bioanalysis of samples from preclinical^2^ and multiple clinical studies.^19−22^

**Figure 5:**
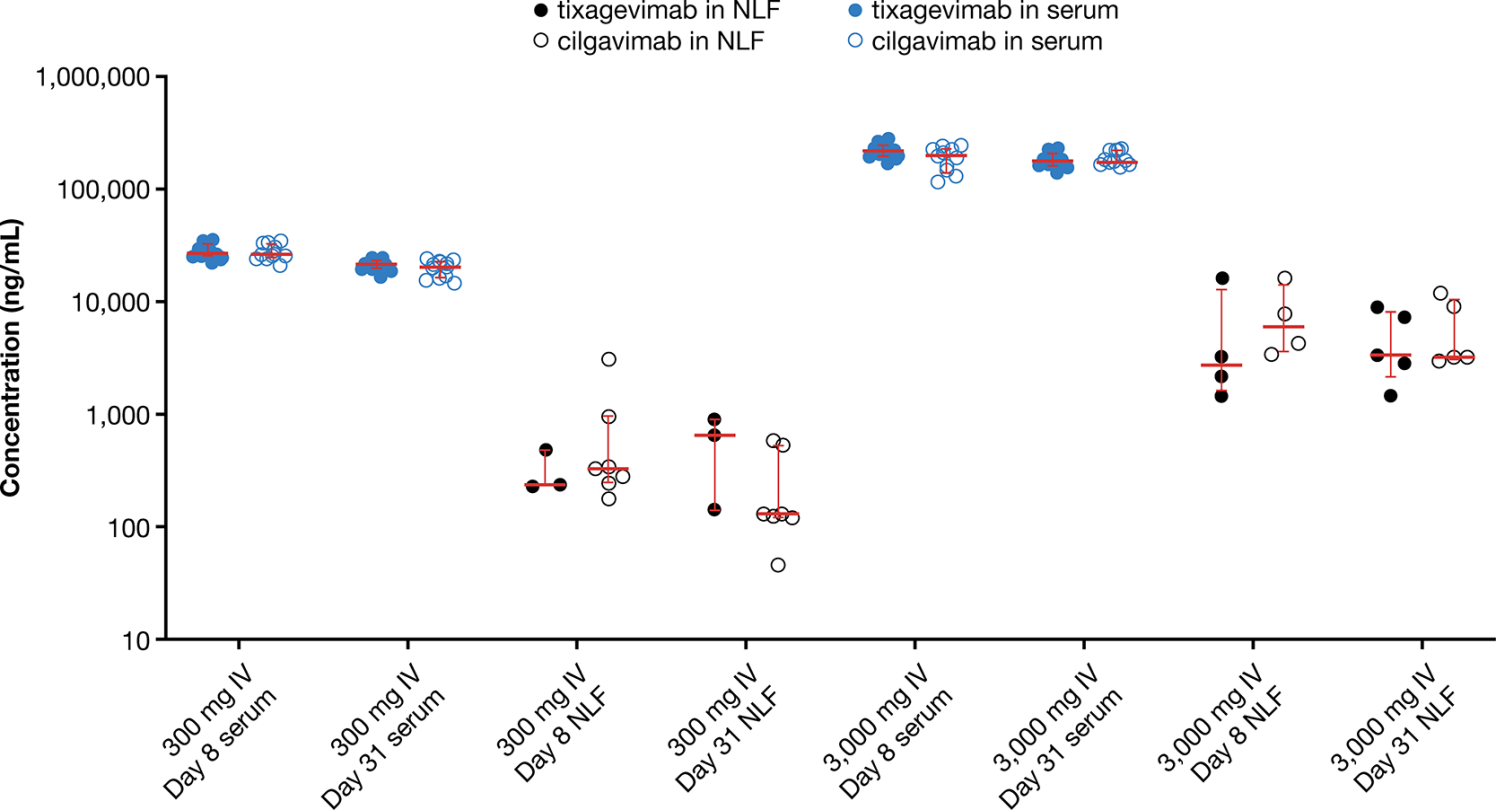
Representative pharmacokinetic (mAb concentration) data in NLF and serum. Error bars indicate median with interquartile range.

Representative chromatograms for tixagevimab and cilgavimab quantification from clinical samples are shown in Figure S1. Representative PK data in human serum and NLF for AZD7442 dosed at 300 and 3,000 mg intravenously are shown in Figure 5.

## Discussion

The unique challenges of addressing bioanalytical needs in support of AZD7442, a COVID-19 mAb combination therapy, enabled development of a robust and highly sensitive method for the quantification of codosed antibodies in serum and NLF, a rare matrix. Typically, the quantification of mAbs can be achieved using LBAs, which require specific reagents (anti-ID) to distinguish between codosed drugs binding to the same target and, in the case of clinical studies, the plethora of human IgGs in circulation.^4,5^ However, these reagents were not readily available and usually take months to generate. More generic capture reagents, such as antihuman Fc antibodies, protein A/G that bind to mAbs, and antigen RBD, would not have differentiated between cilgavimab and tixagevimab, neither as capture reagents nor as detection antibodies for LBA. Previously, a generic capture approach employing protein G coupled to LC-MS/MS-based detection of prototypic peptides emanating from CDRs was successfully employed for the quantification of an antibody–drug conjugate and associated catabolites.^23^ However, this approach required extensive method development (e.g., sequential elution of light chain then heavy chain from the beads) and took many months of method development to achieve the desired 50 ng/mL sensitivity. Recently, a method for quantification of two codosed antibodies REGEN-COV (REGN10933 plus REGN10987, also referred to as casirivimab and imdevimab, respectively) was described,^24^ where a direct digestion approach with trypsin and rAspN was used, followed by LC-MS/MS detection of peptides derived from CDR regions of the REGEN-COV components. The quantification range for REGN-COV in human serum was 10–2,000 μg/mL. Notably, typical sensitivity for LBA-LC-MS/MS quantification of antibodies in circulation using 1D chromatography and analytical flow regime has been reported to be ∼100 ng/mL.^25−28^ Implementation of 2D chromatography,^28−31^ low flow regimes,^32,33^ and antipeptide^34^ can lead to further sensitivity improvements. However, more complex methods inherently suffer from method robustness and cost limitations in applications to large-scale analysis required for clinical trials that frequently involve numerous (>10,000) samples across multiple instruments, analysis batches, and bioanalytical sites over many months and even years.

To meet the sensitivity requirements for the quantification of AZD7442, we developed and validated a highly efficient, robust, sensitive, and selective hybrid LBA-LC-MS/MS assay capable of distinguishing two coadministered mAbs in human serum, and a fit-for-purpose assay for quantification from NLF, based on distinct CDR peptide sequences and capture using the RBD ligand. Assay sensitivity in NLF was 5 ng/mL, which is ∼20× higher compared with typical assays using LBA-LC-MS/MS.

The NLF is a routinely evaluated sample matrix with analytes of interest, including small molecules (e.g., urea, monic acid A), lipid mediators, nucleic acids, and various endogenous peptides. However, analyses of drug concentrations in NLF have been hindered by a lack of convenient, reliable sampling methods (e.g., swabs or nasal lavage) that yield low analyte concentrations which, in turn, produce broad reported ranges for the partition ratio of biopharmaceuticals into the nasopharynx. Common methods for mAb bioanalysis in NLF consist of ELISA,^10,35−37^ electrochemiluminescence,^38,39^ bead-based antibody assays,^40,41^ and other commercially available serological assays.^42^ Generally, the ELISA assays were semiquantitative,^36,37^ intended for research purposes only (with manufacturer reported range of 250–16,000 pg/mL),^35^ or reported standard curve in the ng/mL range (0.195–200 ng/mL).^10^ The sensitivity of the bead-based antibody assays varied by analyte with the lower limit of quantification [LLOQ] for interleukin [IL]-5 being 0.27 pg/mL^41^ and the lower limit of detection [LLOD] being 2.0 pg/mL for IL-5.^40^ For the serological assays the reported LLODs were in the range 0.44–11.00 binding antibody units (BAU/mL),^42^ while for the electro-chemiluminescent assays the reported total protein levels were only in the μg/mL range.^38^ To our knowledge, only one LC-MS/MS method related to protein profiling in NLF has been reported in peer-reviewed publications to date. Gangnus et al. (2022) recently reported NLF kinin peptide quantification using a fully validated LC-MS/MS method employing solid-phase extraction with urea normalization.^43^ The assay achieved high sensitivity with reported LLOQ for kinin peptide quantification in NLF of 1.9 pmol/L. Therefore, the approach reported herein is the first description of quantitative biotherapeutic bioanalysis in NLF using a hybrid LBA-LC-MS/MS method with concentration normalization via urea correlation (described elsewhere).^12,13,44^

In order to ensure the method was applicable to a very broad patient population, we carefully evaluated various factors that could contribute to interferences and matrix effects. Method performance was not affected by the presence of soluble RBD capture reagent to analyte ratios of up to 9-fold, thus ensuring sufficient capture capacity. In addition, we found no apparent interference from codosed mAbs over a 5-fold range in concentrations, thus ensuring that the two mAbs do not interfere with each other’s quantification. Furthermore, in order to assess whether COVID-19 infection status could impact the quantification using RBD-capture method, we calculated that at peak viremia of 10^8^ copies/mL,^45^ with virions expressing an estimated 100 spike proteins,^46^ a maximum RBD concentration of 1.61 ng/mL would be present in infected patient sera. In this assay, bead-bound RBD is used at a concentration of 80 μg/mL for drug capture, over 40,000-fold higher than expected in natural infections, denoting that the potential impact due to competition from viral RBD would be negligible.

Experiments comparing RBD and anti-TM as a capture reagent for tixagevimab and cilgavimab in convalescent and vaccinated sera demonstrated comparable recovery of the analytes at different concentrations and various levels of endogenous anti-SARS-CoV-2 antibody titers. Soluble RBD titration results showed the method quantification accuracy can only be impacted at supra physiologically relevant levels of RBD. Other capture methods, such as anti-YTE, carry a potential risk of interference from antidrug antibodies directed to this modification, with altered PK and loss of protection.^47^

Intravenous and intramuscular methods can result in measurable concentrations of the biotherapeutics in the NLF.^2^ Herein, we used minimally invasive nasosorption strips to collect samples for the assessment of drug concentration in the nasopharynx.^11^ The NLF PK assay was applied to samples collected with nasosorption strips and qualified to measure AZD7442 concentrations in NLF eluates over a very broad quantification range with high recovery. The assay required a high sensitivity and wide dynamic range due to potentially low drug concentration in NLF, variable sample volume, and limited availability of matrix (only one NLF sample was collected per patient per time point). Handling of nasal strips during sample elution was a key challenge for robust analysis, attainment of maximum recovery, and avoidance of sample loss. Urea concentrations in human NLF and serum are equivalent, indicating that urea concentration in NLF provides a useful estimate of NLF volume.^13^ NLF and serum samples were therefore used as a normalization method to account for variability in sample dilutions. Further developments of similar bioanalytical methodology can enable more patient-centric clinical trial designs. Effective implementation of the strategy using hybrid LBA-LC-MS/MS^23,24^ for the quantification of AZD7442 could be expanded to future single/combined mAbs using analogous methods as a powerful alternative to the LBA bioanalytical strategy approach for drug quantification.

## Conclusion

A hybrid LBA-LC-MS/MS method for the absolute quantification of tixagevimab and cilgavimab individually in human and cynomolgus monkey serum was rapidly developed, validated, and implemented. The resulting multiplexed hybrid assay was capable of absolute quantification of the two mAbs individually, demonstrating the feasibility of PK measurements in the absence of anti-ID antibodies with great sensitivity and robustness. Similarly, a highly sensitive LBA-LC-MS/MS-based assay was developed and qualified for the absolute quantification of the two mAbs in human NLF. NLF is a hitherto under-evaluated matrix gaining interest as a patient-centric matrix in studies of COVID-19,^2,37,42,48^ and other respiratory diseases.^49−52^

This approach highlights the importance of mature technology, and an appropriate development strategy to enable the fast deployment of a high-quality PK assay that allowed for the rapid evaluation of AZD7442 during a global COVID-19 pandemic. The application of LBA-LC-MS/MS provides an innovative approach toward the reliable quantification of combination antibody therapy.

## Supporting Information Available

Table S1 (Page S9): Heat Inactivation Results. Table S2 (Pages S10–S11): (a) RBD Capacity Evaluation and (b) Disproportionate Quality Control Evaluation in Human Serum. Table S3 (Pages S12–S13): Human NLF Assay Evaluations (a) Surrogate Matrix Effect and (b) Extraction Recovery. Table S4 (Page S14): Gradient Separation ABI SCIEX API 6500+ (Human Serum and NLF). Table S5 (Page S15): MS/MS Parameters. Table S6 (Page S16): Ionization Source Parameters. Figure S1 (Pages S17–S19): Representative chromatograms for (a) tixagevimab in serum, (b) cilgavimab in serum, (c) tixagevimab in NLF, and (d) cilgavimab in NLF. (PDF)
