# Supplementary material for: Multiplex Hybrid Antigen-Capture LC-MRM Quantification in Sera and Nasal Lining Fluid of AZD7442, a SARS-CoV-2-Targeting Antibody Combination
Source: Anal Chem. 2022 Oct 21;94(43):14835–45. doi: 10.1021/acs.analchem.2c01320 (PMC9631352; doi:10.1021/acs.analchem.2c01320)
Supplement: Supplementary file 1 — ac2c01320_si_001.pdf [file ac2c01320_si_001.pdf]

https://doi.org/10.1021/acs.analchem.2c01320

Supporting Information

Multiplex Hybrid Antigen-Capture LC-MRM Quantification in Sera and Nasal Lining Fluid of AZD7442, a SARS-CoV-2-Targeting Antibody Combination

Ruipeng Mu,[1] Yue Huang,[1,ǁ,*] Jerome Bouquet,[1] Jiaqi Yuan,[1] Robert J. Kubiak,[2] Eric Ma,[3] Sami Naser,[3] William R. Mylott Jr.,[3] Omnia A. Ismaiel,[3,4] Aaron M. Wheeler,[3] Rebecca Burkart,[3] Diego F. Cortes,[3] James Bruton,[3] Rosalinda H. Arends,[2] Meina Liang,[1] and Anton I. Rosenbaum[1,ǁ,*]

[1]*Integrated Bioanalysis, Clinical Pharmacology and Safety Sciences, R&D, AstraZeneca, South San Francisco, CA 94080, USA*

[2]*Clinical Pharmacology and Quantitative Pharmacology, Clinical Pharmacology and Safety Sciences, R&D, AstraZeneca, Gaithersburg, MD 20878, USA*

[3]*Research and Development Biologics by LC-MS/MS, PPD Laboratories, Richmond, VA 23230, USA*

[4]*Faculty of Pharmacy, Zagazig University, Zagazig 2, Egypt*

[ǁ]These authors contributed equally to this work

***Corresponding Authors**

Anton I. Rosenbaum - *121 Oyster Point Blvd, South San Francisco, CA 94080, USA*; Tel: +1-650-379-3099; E-mail: anton.rosenbaum@astrazeneca.com

https://doi.org/10.1021/acs.analchem.2c01320

Yue Huang - *121 Oyster Point Blvd, South San Francisco, CA 94080, USA*; Tel: +1-650-760-0510; E-mail: yue.huang@astrazeneca.com

Six tables, one figure; 19 pages.

Table S1 (Page S9): Heat Inactivation Results.

Table S2 (Page S10–S11): (a) RBD Capacity Evaluation and (b) Disproportionate Quality Control Evaluation in Human Serum.

Table S3 (Page S12–S13): Human NLF Assay Evaluations (a) Surrogate Matrix Effect and (b) Extraction Recovery.

Table S4 (Page S14): Gradient Separation ABI SCIEX API 6500+ (Human Serum and NLF).

Table S5 (Page S15): MS/MS Parameters.

Table S6 (Page S16): Ionization Source Parameters.

Figure S1 (Page S17–S19): Representative chromatograms for (a) tixagevimab in serum, (b) cilgavimab in serum, (c) tixagevimab in NLF, and (d) cilgavimab in NLF.

https://doi.org/10.1021/acs.analchem.2c01320

Supporting Experimental Section

Hybrid LBA-LC-MS/MS

Materials: Tixagevimab and cilgavimab reference standards were provided by AstraZeneca (Gaithersburg, MD, USA). Stable isotope labeled internal standard peptides, ASGF and DVWM, were supplied by Elim Biopharmaceuticals (Hayward, CA, USA). Receptor-binding domain (RBD) of severe acute respiratory syndrome coronavirus 2 (SARS-CoV-2) and anti-TM antibody were provided by AstraZeneca (Gaithersburg, MD, USA). Both the RBD protein and anti-TM antibody were biotinylated using a biotinylation kit (EZ-Link™ Sulfo-NHS-LC-Biotin, Thermo Fisher Scientific [Waltham, MA, USA]). Cynomolgus monkey and human sera were purchased from BioIVT (Hicksville, NY, USA). High-grade water was obtained using a Milli-Q internal water purification system. Bovine serum albumin (BSA), DTT, dimethyl sulfoxide, ethanol, formic acid, iodoacetamide, and isopropanol were obtained from Sigma-Aldrich (St. Louis, MO, USA). Acetonitrile, acetonitrile with formic acid, ammonium bicarbonate, methanol, and 6 N hydrochloric acid were purchased from VWR Scientific (Allison Park, PA, USA). RapiGest™ SF surfactant was purchased from Waters (Milford, MA, USA). Tris(hydroxymethyl)aminomethane-buffered saline (TBS) and TBS-Tween-20 (TBST) were obtained from Boston Bioproducts (Ashland, MA, USA). High-capacity Magne® streptavidin beads were purchased from Promega Corporation (Madison, WI, USA). Pierce™ Trypsin Protease (MS grade) was purchased from Thermo Fisher Scientific (Waltham, MA, USA). Dulbecco's phosphate-buffered saline (PBS) was purchased from Mediatech (Manassas, VA, USA).

Instrumentation: To increase efficiency, the same LC equipment and conditions were used for the assays of cynomolgus monkey serum (RBD capture), human serum (RBD and anti-TM antibody capture), and human nasal lining fluid (NLF; RBD capture). An LC PAL autosampler with DLW was used from CTC Analytics AG (Zwingen, Switzerland). LC pumps comprised Agilent 1260 Infinity II and 1100 series pumps, and an Agilent 1290 Infinity II ACH column heater was used (Agilent, Santa Clara, CA, USA). A SCIEX API 5000 triple quadrupole mass spectrometer was used for the cynomolgus monkey serum assay, and a SCIEX API 6500+ triple quadrupole mass spectrometer was used for the human serum and NLF assays (Framingham, MA, USA). Magnetic beads were processed using a KingFisher Flex System from Thermo Fisher Scientific (Waltham, MA, USA). Peak area integration and calculations of sample concentrations were performed using SCIEX Analyst^{TM} software, version 1.6.2 (Framingham, MA, JUSA) and PPD Assist LIMS, version 6 (Richmond, VA).

Serum assays: The methodology surrounding the identification and quantification of tixagevimab and cilgavimab in serum is illustrated in **Figure 1a**. Briefly, a human serum sample (20 µL) and TBST buffer with 1% BSA (100 µL) were mixed thoroughly before adding 25 µL of washed streptavidin magnetic beads coated with biotinylated RBD protein (0.08 mg RBD protein/mL bead). After capture for 2 h at room temperature (RT) with constant shaking, a TBS-based buffer was used to wash the beads thoroughly; after 2h RT capture, the beads could instead be incubated at 2–8 °C, with constant mixing, overnight for up to 18 h, followed by the bead washing steps. The overnight capture option (1 day vs 2 days) provided for procedural flexibility. After washing with the KingFisher Flex System, the beads were transferred

to the elution plate with a digestion buffer consisting of 10:20:70 methanol/1.0 M ammonium bicarbonate/water (v/v/v), RapiGest^{TM} solution (0.025%), and 0.010 mL of 0.1 M DTT, followed by incubation at 65 °C for 1 h. After reduction, alkylation was performed by adding 0.025 mL of 0.1 M iodoacetamide to the elution plate and incubating at RT for 30 min, protected from light, with no need for quenching of iodoacetamide. Digestion was performed after alkylation by adding trypsin solution (2.5 µg) and incubating at 37 °C for 2 h. After digestion, internal standard working solution containing stable isotope labeled peptides (ASGF-IS/DVWM-IS, 50/50 ng/mL) was added to the samples. Trypsin digestion was quenched by adding 10 µL of 2 N hydrochloric acid. The contents of the elution plates were passed through a Multiscreen HTS Filter Plate (Sigma-Aldrich; St. Louis, MO, USA) into the injection plates. The human serum assay using anti-TM antibody for capture followed the same steps as the human serum assay using RBD for capture, except for using 25 µL of washed streptavidin magnetic beads coated with biotinylated anti-TM antibody (0.5 mg anti-TM antibody/mL bead). The cynomolgus monkey serum assay, which used RBD for capture, followed the same steps as the human serum assay using RBD for capture, except the sample aliquot was diluted 10-fold with TBST buffer and 1% BSA, prior to adding the streptavidin magnetic beads coated with biotinylated RBD protein (dilution was performed due to assay range differences between cynomolgus monkey and human serum).

NLF assay: For the human NLF assay, patient samples were collected using a noninvasive Nasosorption^{TM} FXi nasal sampling device (Mucosal Diagnostics, Midhurst, UK), which uses a synthetic absorptive matrix swab to absorb NLF from the nasal mucosa (**Figure 1b**). The moist strip is removed from the device and

added to 300 µL of elution buffer (1X PBS pH 7.4, 1 mg/mL BSA, and 1% NP-40 Surfact-AmpsTM [Thermo Fisher Scientific, Waltham, MA, USA]). Due to the limited collection volume and availability of blank NLF, a surrogate matrix consisting of 2.5% human serum, 1X PBS pH 7.4, 1 mg/mL BSA, and 1% NP-40 was used for preparing calibration standards and quality control samples. Human NLF eluant sample (180 µL) was added to 25 µL of washed streptavidin magnetic beads coated with biotinylated RBD protein. The remaining processing steps were identical to the human serum steps (RBD capture) described above. Due to expected variability in NLF volumes from patients and in extraction efficiency of the nasal swabs, urea concentrations were determined and used to correct/normalize the tixagevimab and cilgavimab concentrations in human NLF. Urea concentrations were determined using a urea assay kit (Abcam, Cambridge, UK), following manufacturer's instructions over a concentration range of 1.2–6.0 µg/mL. Briefly, human NLF eluant sample (5 µL) was added to 45 µL of urea assay buffer (kit-supplied). To each sample, 50 µL of reaction mix (kit-supplied, consisting of urea assay buffer, OxiRed prob, enzyme mix, developer, and convertor enzyme) was added and vortexed for 2 min at RT; immediately followed by incubation at 37 °C for 1 h, protected from light. Analysis was performed on a SprectraMAX M3 microplate reader, optical density 570 nm (Molecular Devices, San Jose, CA).

Serological Methods – Anti–SARS-CoV-2 Antibody Titers

Anti–SARS-CoV-2 IgG, immunoglobulin M (IgM), and immunoglobulin A (IgA) antibody titers in human serum were measured using Meso Scale Discovery electrochemiluminescence methods validated following best practices. SARS-CoV-2 spike RBD was coated as a capture reagent for each method, and reactive IgG, IgM,

and IgA were detected using detection antibodies specific to their respective immunoglobulin class. The amount of bound detection reagent was detected by a chemiluminescent signal generated when a voltage was applied. The resulting signal is proportional to the amount of reactive IgG, IgM, or IgA present in the serum sample. Signals for each sample on the coated half of the plate were corrected by background subtraction of the signal on the uncoated half of the plate. The resulting normalized data were used to determine sample concentration by interpolation from the positive control curve.

LC-MS/MS conditions: As described above, two tryptic peptides, ASGF and DVWM, were monitored in this assay for the measurement of tixagevimab and cilgavimab, respectively. The chromatographic separation, consistently applied across each of the methods, was performed on a Waters ACQUITY UPLC HSS T3 C18, 2.1 × 50 mm, 1.8 µm column, maintained at 45 °C, with 0.1% formic acid in water as mobile phase A, 0.1% formic acid in acetonitrile as mobile phase B, and an additional “make-up” mobile phase C (MPC) of 0.1% formic acid in 50:50 acetonitrile/water. The gradient separation used for human serum and NLF samples is described in **Table S4**. To reduce potential for ion optics contamination, an Agilent 1290 Infinity II ACH switching valve was employed, allowing the column eluent to be directed to the mass spectrometer from 1.2–2.3 min, with “make-up” MPC flowing to the mass spectrometer at all other times. Retention times of cilgavimab and tixagevimab tryptic peptides were 1.6 and 2.0 min, respectively, with a total run time of 5 min.

Mass spectrometric detection was carried out using AB SCIEX API 6500+ (human serum and NLF) and AB SCIEX API 5000 (cynomolgus macaque serum) triple quadrupole mass spectrometers (AB SCIEX, Framingham, MA, USA). Each mass spectrometer was equipped with a TurboIonSpray® source and operated in positive ionization mode, using multiple reaction monitoring (MRM; **Tables S5 and S6**). Data were acquired using Analyst, version 1.6.2 (AB Sciex, Framingham, MA, USA). A linear, 1/concentration^2 weighted, least squares regression algorithm was used to quantify unknown samples.

https://doi.org/10.1021/acs.analchem.2c01320

Table S1. Heat Inactivation Results

**Concentration in plasma (µg/mL)**		**Peak area ratio of the heat inactivated sample/peak area ratio of the control**						
		**DVWM from cilgavimab**			**ASGF from mAb tixagevimab**			
Cilgavimab	Tixagevimab	540.13/865.04	540.13/678.83	540.13/433.02	837.96/1,312.53	837.96/1,211.42	837.96/1,064.25	837.96/933.05
1	1	90%	86%	86%	117%	108%	122%	132%
10	10	87%	74%	83%	107%	124%	131%	143%
100	100	83%	48%	80%	182%	110%	147%	57%
1	10	78%	88%	81%	141%	154%	109%	66%
10	100	82%	75%	78%	121%	112%	107%	104%
10	1	73%	62%	75%	101%	97%	84%	121%
100	10	80%	86%	75%	68%	92%	108%	90%

Experiment detail: Recovery (%) upon heat inactivation (60 °C for 60 min) was assessed. Samples were prepared at 1:1, 1:10, and 10:1 concentration in pooled human plasma. The plasma was then split to two aliquots. One aliquot was incubated on heat block (with water) for 1 h then cooled to RT. The other aliquot was kept on ice. Cilgavimab in general had a decrease after heat inactivation. Tixagevimab increased slightly. Capture was performed for 1.5 h at 37 °C.

ASGF and DVWM are proteotypic peptides with ASGFTFMSSAVQWVR and DVWMSWVR sequences, respectively.

mAb, monoclonal antibody; RT, room temperature.

https://doi.org/10.1021/acs.analchem.2c01320

Table S2. (a) RBD Capture Capacity Evaluation and (b) Disproportionate Quality Control Evaluation in Human Serum

(a)

Group	Beads volume (RBD/analyte ratio)	Theoretical concentration (μg/mL)	Tixagevimab		Cilgavimab	
			Calculated concentration (μg/mL)	Accuracy (%)	Calculated concentration (μg/mL)	Accuracy (%)
A	10 μL (~3.6×)	30	33.1	110.3	34.1	113.7
			33.5	111.7	34.4	114.7
			31.4	104.7	33.0	110.0
B	15 μL (~5.4×)	30	32.8	109.3	32.7	109.0
			33.8	112.7	33.5	111.7
			34.4	114.7	34.2	114.0
C	25 μL (~9×)	30	28.9	96.3	29.0	96.7
			33.4	111.3	31.6	105.3
			28.8	96.0	29.6	98.7
			33.1	110.3	31.0	103.3

https://doi.org/10.1021/acs.analchem.2c01320

(b)

**Ratio of Disproportionate QC**	**Cilgavimab : Tixagevimab = 1:5**		**Cilgavimab : Tixagevimab = 5:1**	
**Analyte**	**Cilgavimab**	**Tixagevimab**	**Cilgavimab**	**Tixagevimab**
**Theoretical concentration (µg/mL)**	**4.5**	**22.5**	**22.5**	**4.5**
**Calculated concentration (µg/mL)**	4.55	22.9	20.8	4.19
	4.40	20.0	21.0	4.30
	3.99	22.2	19.0	4.23
	4.05	20.7	19.8	4.25
	4.32	20.8	22.6	4.09
	4.04	21.1	20.2	4.47
**Mean percent difference from theoretical**	-6.09%	-5.26%	-8.58	-5.46%
**CV**	5.42%	5.04%	6.07%	2.96%

CV, coefficient of variation; RBD, receptor-binding domain.

https://doi.org/10.1021/acs.analchem.2c01320

Table S3. Human NLF Assay Evaluations (a) Surrogate Matrix Effect and (b) Extraction Recovery

(a)

	**0% pooled human serum**				**5% pooled human serum**			
	**QC 1**		**QC 2**		**QC 1**		**QC 2**	
	**Cilgavimab**	**Tixagevimab**	**Cilgavimab**	**Tixagevimab**	**Cilgavimab**	**Tixagevimab**	**Cilgavimab**	**Tixagevimab**
Theoretical Conc. (ng/mL)	15	15	1,150	1,150	15	15	1,150	1,150
Calculated Conc. (ng/mL)	16.9	15.0	1,380	1,150	16.4	17.0	1,180	1,330
	18.2	16.2	1,380	1,140	16.8	17.9	1,200	1,360
	18.1	16.9	1,440	1,200	15.9	14.6	1,270	1,380
Mean % difference from theoretical	18.2	6.97	21.5	10.0	8.97	1.33	5.84	17.9
Mean % difference from 0% pooled HuSe control	N/A				–7.91	3.13	–12.9	16.20

(b)

	**Cilgavimab**		**Tixagevimab**	
Theoretical concentration (ng/mL)	15.0	1,150	15.0	1,150
Recovery (%)	93.4	101.7	96.8	89.9
	92.4	99.0	103.5	85.6
	105.3	91.6	90.6	86.7
Mean recovery (%)	97.0	97.5	97.0	87.4
CV	7.4%	5.4%	6.7%	2.6%
Overall recovery (%)	97.2%		92.2%	

CV, coefficient of variation; HuSe, human serum; N/A, not applicable; NLF, nasal lining fluid; QC, quality control.

https://doi.org/10.1021/acs.analchem.2c01320

Table S4. Gradient Separation ABI SCIEX API 6500+ (Human Serum and NLF)

Time (min)	Flow rate (mL/min)	0.1% formic acid in water (%A)	0.1% formic acid in acetonitrile (%B)
0.00	0.35	76	24
0.20	0.35	76	24
2.60	0.35	59	41
2.61	0.45	5	95
4.00	0.45	5	95
4.10	0.45	76	24
5.00	0.45	76	24

NLF, nasal lining fluid.

https://doi.org/10.1021/acs.analchem.2c01320

Table S5. MS/MS Parameters

Mass spectrometer (samples)	Analyte	TR (min)	Dwell time (ms)	Q1 (m/z)	Q3 (m/z)	DP (V)	CE (V)	CXP (V)	EP (V)
ABI SCIEX API 6500+ (human serum and NLF)	DVMW-IS	1.75	50	544.9	874.4	40	30	16	10
	DVWM	1.75	50	539.9	864.4	40	30	16	10
	ASGF-IS	2.16	50	842.6	942.5	80	42	16	10
	ASGF	2.16	50	837.6	932.5	80	42	16	10
ABI SCIEX API 5000 (cynomolgus macaque serum)	DVMW-IS	2.34	25	544.9	874.6	80	29	29	10
	DVWM	2.34	35	539.9	864.6	80	29	29	10
	ASGF-IS	2.67	25	843.1	942.5	100	40	33	10
	ASGF	2.67	35	838.0	932.6	100	40	33	10

ASGF, surrogate for tixagevimab; CE, collision energy; CXP, collision cell exit potential; DP, declustering potential; DVWM, surrogate for cilgavimab; EP, entrance potential; IS, internal standard; MS/MS, tandem mass spectrometry; NLF, nasal lining fluid; Q, quartile; TR, retention time.

ASGF and DVWM are proteotypic peptides with ASGFTFMSSAVQWVR and DVWMSWVR sequences, respectively.

https://doi.org/10.1021/acs.analchem.2c01320

Table S6. Ionization Source Parameters

MS parameter	API 6500+	API 5000
Ion Source Temp (TEM)	550 °C	550 °C
IonSpray Voltage (IS)	5,500 V	5,500 V
Collision Gas Flow (CAD)	10	4
Curtain Gas Flow (CUR)	30	20
Nebulizer Gas Flow (NEB/GS1)	70	65
Turbo IonSpray Gas (AUX/GS2)	65	65
Processing software	Analyst software, version 1.6.2	

IS, internal standard; MS, mass spectrometry.

https://doi.org/10.1021/acs.analchem.2c01320

Figure S1. Representative chromatograms for (a) tixagevimab in serum, (b) cilgavimab in serum, (c) tixagevimab in NLF, and (d) cilgavimab in NLF

a

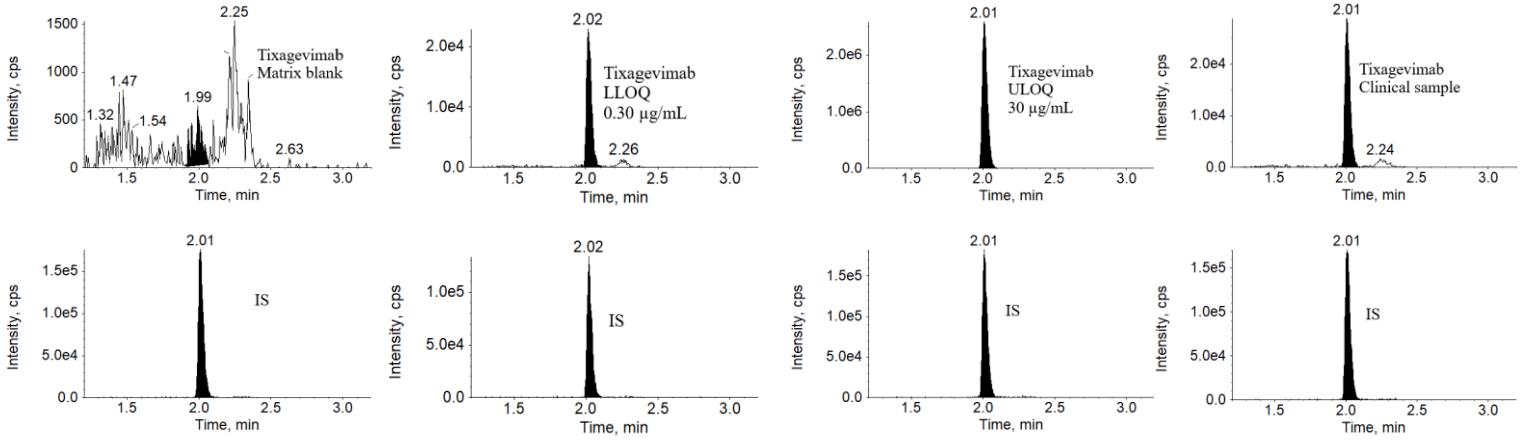

https://doi.org/10.1021/acs.analchem.2c01320

b

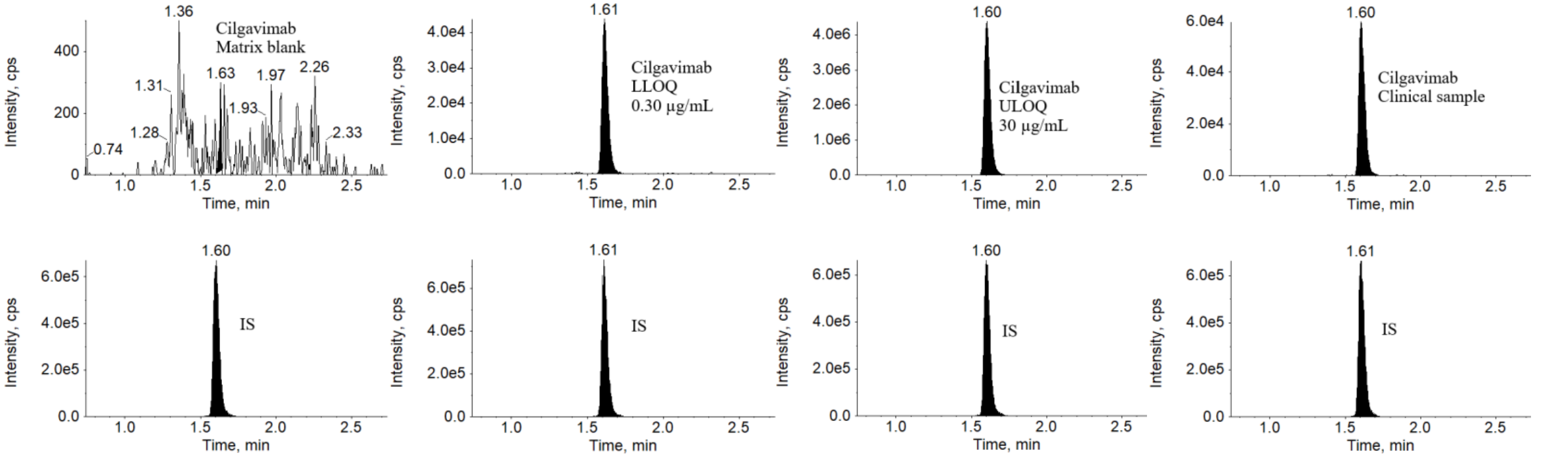

c

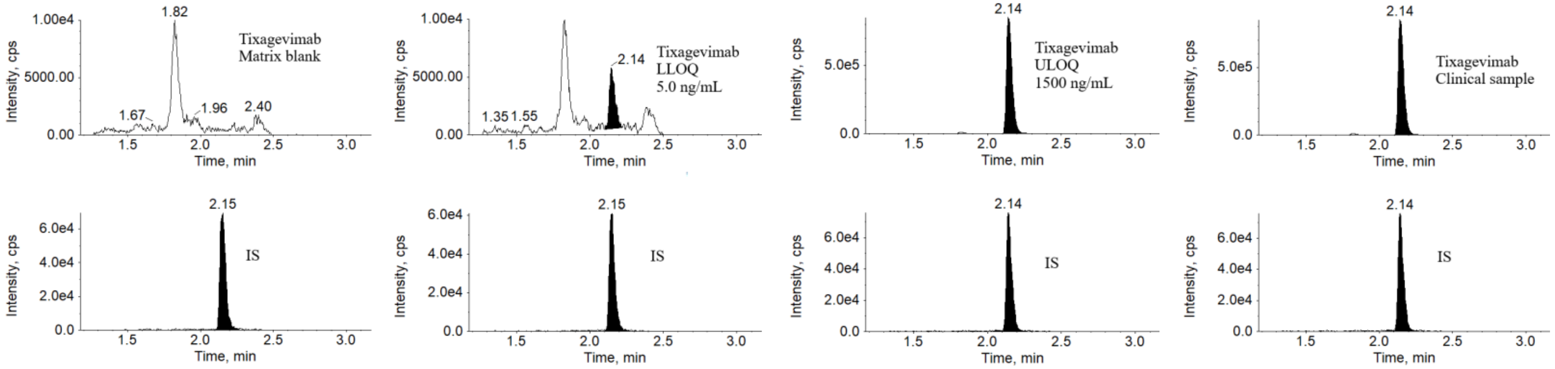

https://doi.org/10.1021/acs.analchem.2c01320

d

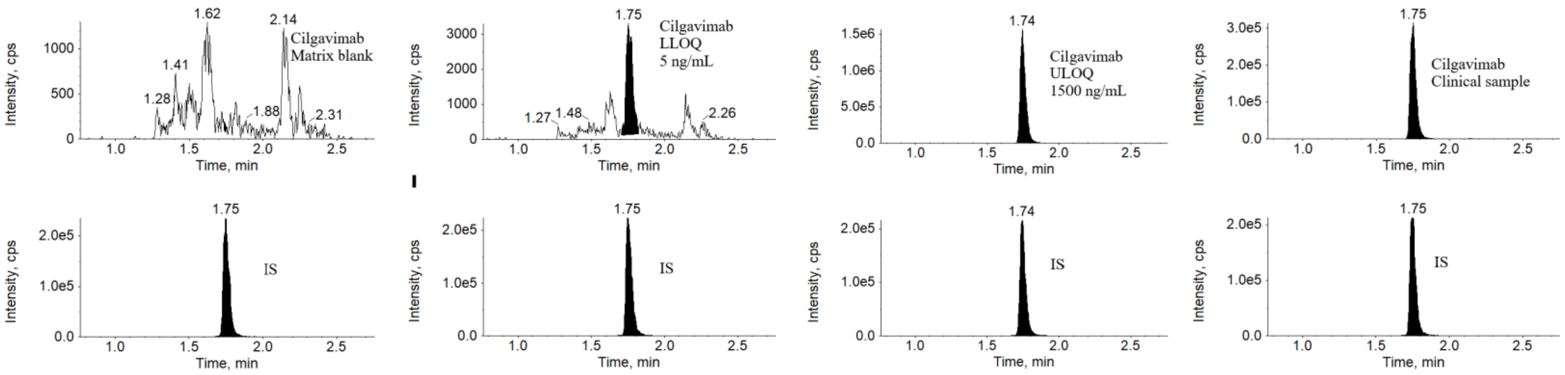

The serum assay method was validated for tixagevimab and cilgavimab absolute quantification in human serum, with a dynamic range for each of 0.3–30 µg/mL. In cynomolgus monkey serum, the dynamic range was 9–1,000 µg/mL. The NLF assay method was qualified for tixagevimab and cilgavimab absolute quantification in eluted solution from nasosorption device, with a dynamic range of 5–1,500 ng/mL. Representative serum clinical sample was taken 8 h following a 300 mg dose, and a representative NLF sample was taken 168 h following a 3,000 mg dose.

cps, counts per second; IS, internal standard; LLOQ, lower limit of quantitation; NLF, nasal lining fluid; ULOQ, upper limit of quantification.
